# The Gas Exhibition. I.—Model Laundry, Cooking Stoves, and Hospital Ranges

**Published:** 1913-10-11

**Authors:** 


### THE GAS EXHIBITION.

### I. Model Laundry, Cooking Stoves, and Hospital Ranges.

THE Gas Exhibition now being held at Shepherd's Bush is a striking example of energy and enterprise stimulated to the tenth degree by urgent competition.

It was the original intention of the Exhibition authorities to make a particular appeal to hospital officers, and for this purpose a special booklet, "Gas for the Hospital," was prepared and published. Further, a model operating theatre and a section of a hospital ward were projected. The theatre, unfortunately, did not materialise, owing to some unforeseen difficulty at the last moment, and the room which should have housed that exhibit now contains merely a number of lavatory basins. It is to be regretted that the opportunity of observing the effect of indirect gas lighting on an operating-table has been lost. The section of a ward, however, has been fitted up.

One of the first matters to attract attention on entrance into the Exhibition is a model laundry, in which gas is used for all the principal processes—ironing, goffering, calendering, etc. The irons are supplied with low-pressure gas by flexible tubing, while the lighting and other machinery is supplied with high-pressure gas. It is worthy of note that Messrs. Spiers and Pond (of whose laundry the exhibit is a section) went very thoroughly into the respective merits of gas and electricity when equipping their works seven years ago. Their choice of gas has passed the test of a subsequent examination of the two systems. The engineer in charge of the laundry states that, apart from other advantages, the cost of renewals and repairs is much lower with gas, since gas irons are much simpler in construction than electric irons. Such a concrete example cannot but be of value to hospitals which have, or contemplate having, their own laundries.

A further noteworthy point about this exhibit is that it uses its own gas compressor, thereby converting the ordinary low-pressure gas into high-pressure. The greatly increased efficiency of the light thus obtained is very remarkable. High-pressure gas can usually be supplied direct from the company's mains in the central districts of London, but in many of the suburbs it is not available, in which case the consumer must use his own compressor if he desires the benefits of high-pressure gas. The price of a compressor (Keith and Blackman, Ltd.), delivering 200 cubic feet per hour, is £9, or £18 for one delivering 500 cubic feet per hour.

Opposite to the model laundry is an exhibit of incandescent-mantle making. While extremely interesting, we allude to it here only to point the moral that upright mantles are now regarded as out of date, the inverted burner being used on practically every point throughout the Exhibition.

It is, perhaps, in the direction of cooking that gas is at present chiefly used in hospitals. The Exhibition offers a valuable assortment of cooking stoves and ranges. The smaller stoves demonstrate all the latest types, and show several useful improvements. One of these is an arrangement (called the "Larkin" boiler), by which a receptacle full of water at the side of the stove becomes heated by the mere use of the inside oven, thus ensuring a supply of warm water without the necessity for lighting the rings. In the hotel kitchen the large range (the "Langham") manufactured by Messrs. Fletcher, Russell and Co. will attract the special attention of the hospital officer. The ovens in the range are heated externally by gas burners fitted underneath the bottom plates, so that no gas is present in the food compartment. Each ring on the top hot-plate has over it a steel plate, which can be removed for cleaning, or for securing a quick boil. The bottoms of the ovens are covered with thick fireclay slabs to increase the heating efficiency. It is interesting to learn that the London Hospital has placed an order for one of these ranges in their new kitchen. The same principle of external heat applies to the smaller roasters of the new type, and these enable the cooking of meat, milk puddings, and pastry to be done simultaneously and with the same consumption of gas as would have been required for each in the older stoves. The utility of gas for cooking is not, however, confined to stoves and ovens, for the same heat-producer is used for vegetable steamers, stock-pots, hot-plates, milk-boilers, grills, fish-friers, and egg-boilers. In the case of hot-plates an automatic burner can be fixed, so that on the utensils being removed the gas goes out. The cost of cooking by gas, based on the experiences of a large London hospital, is stated by the authorities to be as follows :—

Main kitchen ......... 9s. 4d. per bed per annum.
Ward kitchen ........ 12s. 4d. per bed per annum.

[The gas meters, fires, destructors and geysers will be described in a further notice next week.]

